# Rotavirus vaccination and incidence of type 1 diabetes: a population-based natural experiment

**DOI:** 10.1007/s00125-026-06832-0

**Published:** 2026-08-28

**Authors:** Maria Östman, Ketil Størdal, German Tapia, Lars C. Stene

**Affiliations:** 1 Faculty of Medicine, University of Oslo, Oslo, Norway; 2 Department of Paediatric Research, University of Oslo, Oslo, Norway; 3 Department of Paediatric and Adolescent Medicine, Oslo University Hospital, Oslo, Norway; 4 Department of Chronic Diseases, Norwegian Institute of Public Health, Oslo, Norway

**Keywords:** Children, Cohort, Epidemiology, Rotavirus, Rotavirus vaccine, Type 1 diabetes, Vaccination

## Abstract

**Aims/hypothesis:**

Environmental factors, including infections, have been suspected to influence the risk of type 1 diabetes. Studies of rotavirus vaccine and risk of type 1 diabetes have shown conflicting results. We took advantage of the rapid and high uptake of the rotavirus vaccine among infants in Norway as a natural experiment to study the potential association between rotavirus vaccination and type 1 diabetes incidence in Norway.

**Methods:**

In this cohort study, we followed all children born in Norway during 2007–2019 from 6 months to 5 years of age for type 1 diabetes. In an interrupted time series analysis, we compared type 1 diabetes incidence in children born before compared with after introduction of the rotavirus vaccination programme in September 2014.

**Results:**

Of 740,744 included children, 846 were diagnosed with type 1 diabetes between 6 months and 5 years of age. Our primary interrupted time series analysis showed an increasing trend after implementation of the rotavirus vaccination programme (relative HR 1.11 [95% CI 1.03, 1.20]), which was not significant after excluding birth cohorts from 2013–2015 to avoid potential herd immunity effects (relative HR 1.05 [95% CI 0.93, 1.18]). In a secondary analysis comparing fully vaccinated with non-vaccinated children, the adjusted HR was 1.17 (95% CI 0.78, 1.77).

**Conclusions/Interpretation:**

This study found no evidence for a reduced risk of type 1 diabetes after introduction of rotavirus vaccination in infancy.

**Graphical Abstract:**

**Figure:**
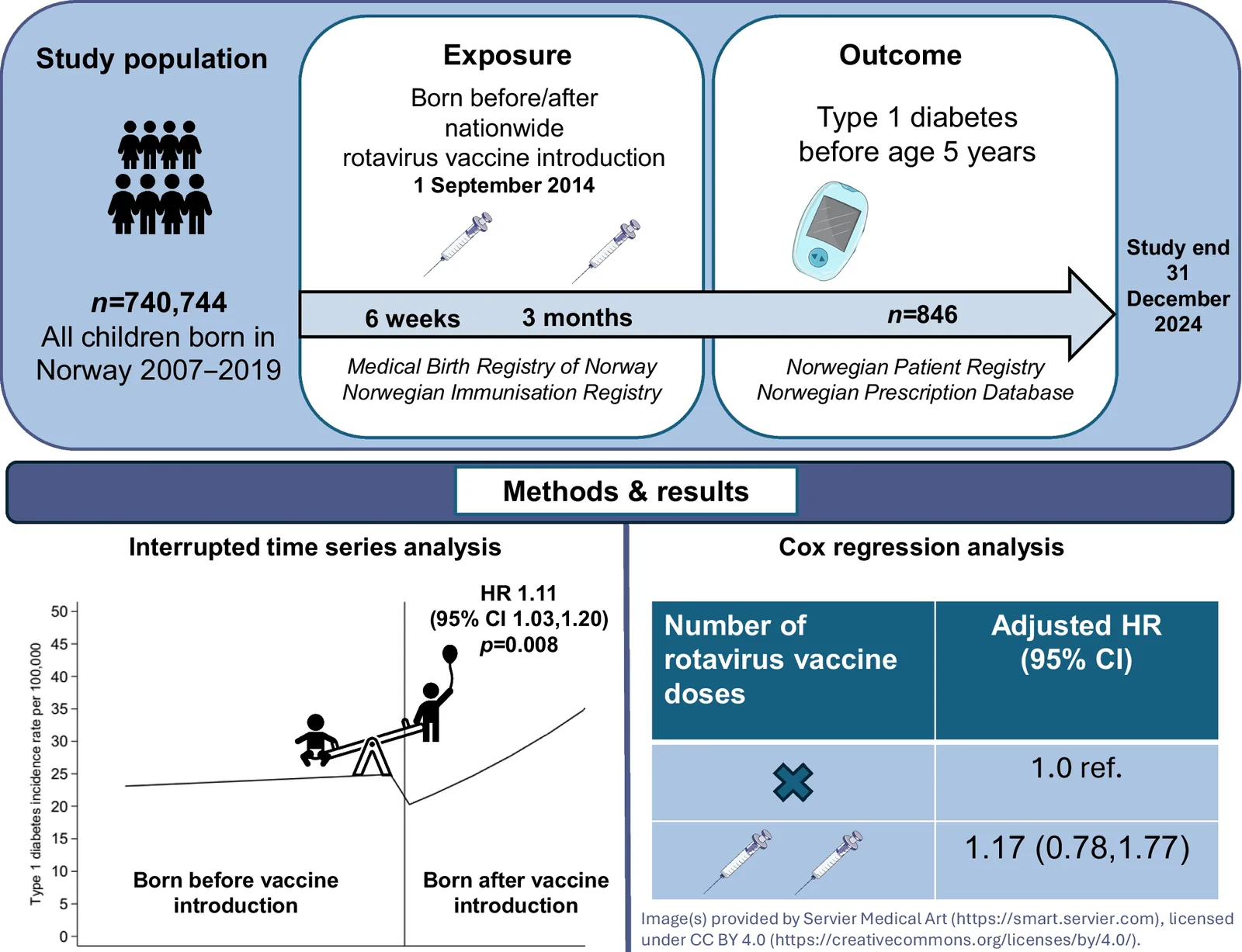

**Supplementary Information:**

The online version contains peer-reviewed but unedited supplementary material available at https://doi.org/10.1007/s00125-026-06832-0.

**Figure:**
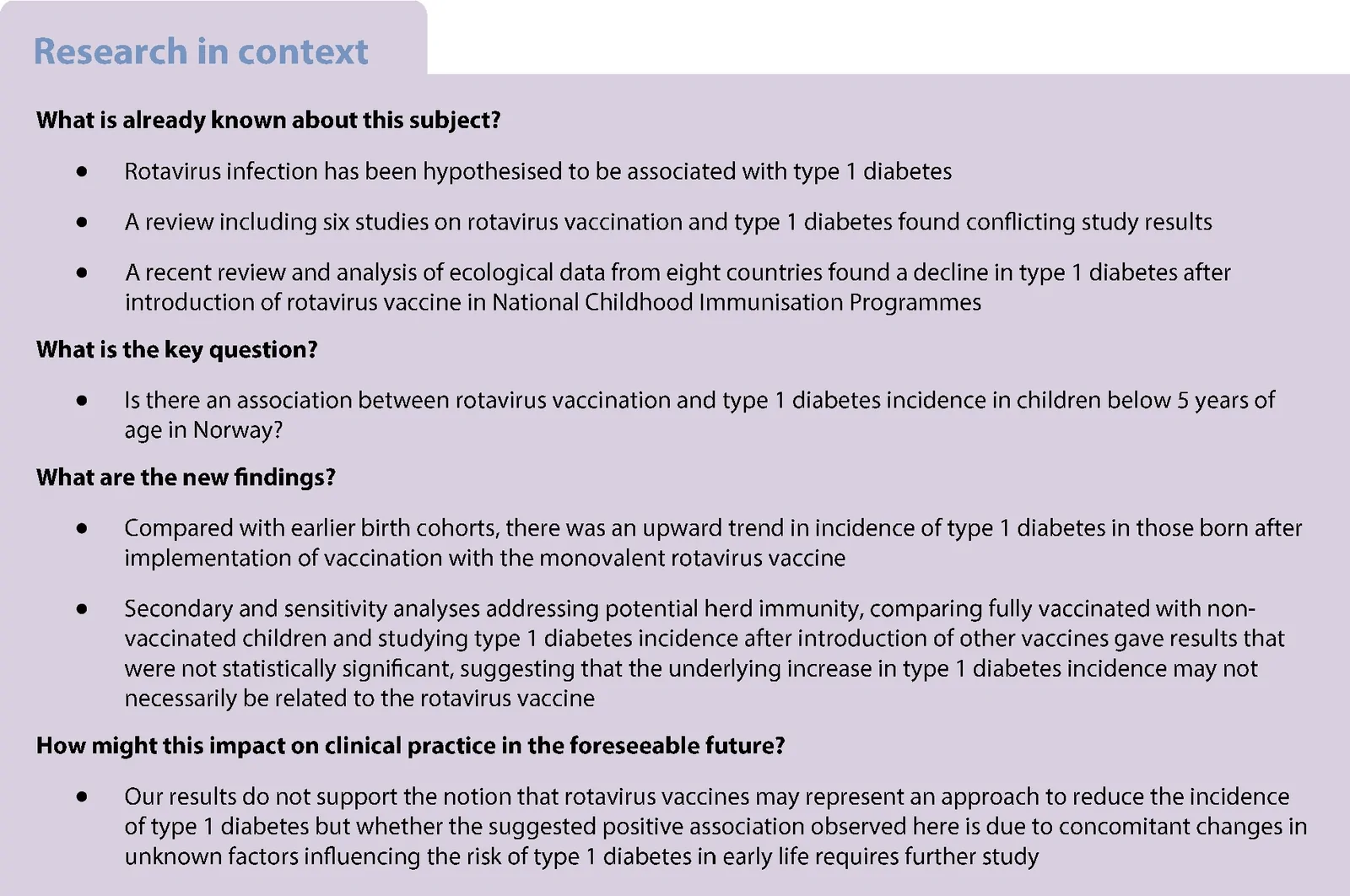

## Introduction

Type 1 diabetes is an autoimmune disease, where loss of insulin-producing beta cells in the pancreas leads to hyperglycaemia requiring life-long insulin treatment. The incidence rate of type 1 diabetes in Norway is among the highest globally, with an estimated incidence in 2025 of 36.8 per 100,000 in children below the age of 15 years [1]. Type 1 diabetes occurs in genetically predisposed individuals and the highest risk is conferred by the combination of HLA DR3-DQ2 and HLA DR4-DQ8 haplotypes [2]. The changing time trends in type 1 diabetes incidence observed among children worldwide over the past decades cannot be explained by genetic changes [3]. Environmental factors, such as infections, diet and environmental chemicals, are believed to contribute to type 1 diabetes pathogenesis [4].

Rotavirus was previously the main causative organism in children under 5 years of age hospitalised for gastroenteritis in Norway and globally [5, 6]. Rotavirus infection has been hypothesised to be associated with type 1 diabetes [7]. Suggested mechanisms of action include molecular mimicry between the virus protein (VP) 7 and beta cell antigen, bystander activation and viral persistence [8]. A Norwegian study found increased risk of type 1 diabetes in children hospitalised with gastroenteritis, regardless of infectious agents, during the first 18 months of life [9], though studies are conflicting [7].

Vaccination with the human attenuated Rotarix monovalent oral rotavirus vaccine (RV1) was introduced in Norway for children born on 1 September 2014 or later in the National Childhood Immunisation Programme. Four years after implementation of the vaccination programme, hospitalisation due to rotavirus infection was reduced by 76% in children under 5 years of age [5]. RV1 is administered orally in two doses, the first at the age of 6 weeks and the second at the age of 3 months [10]. We have previously published an article in the same study sample that found no significant change in the incidence rate of coeliac disease before 5 years of age after introduction of RV1 in the Norwegian Childhood Immunisation Programme [11]. The vaccine coverage in our previously published article was 92.8% for complete vaccination after introduction of the vaccination programme, in contrast to almost no vaccination before its introduction [11]. Many countries use RV1, while others use RotaTeq pentavalent oral rotavirus vaccine (RV5), a reassortant human-bovine rotavirus vaccine. This vaccine is administered orally in three doses [12]. There are also two other WHO prequalified vaccines that are less commonly used, Rotasiil and Rotavac [12].

Studies on rotavirus vaccination and risk of type 1 diabetes have produced conflicting results [7]. We aimed to study whether there is an association between rotavirus vaccine implementation and early-onset type 1 diabetes in a nationwide Norwegian birth cohort.

## Methods

### Study design and setting

**Fig. 1:**
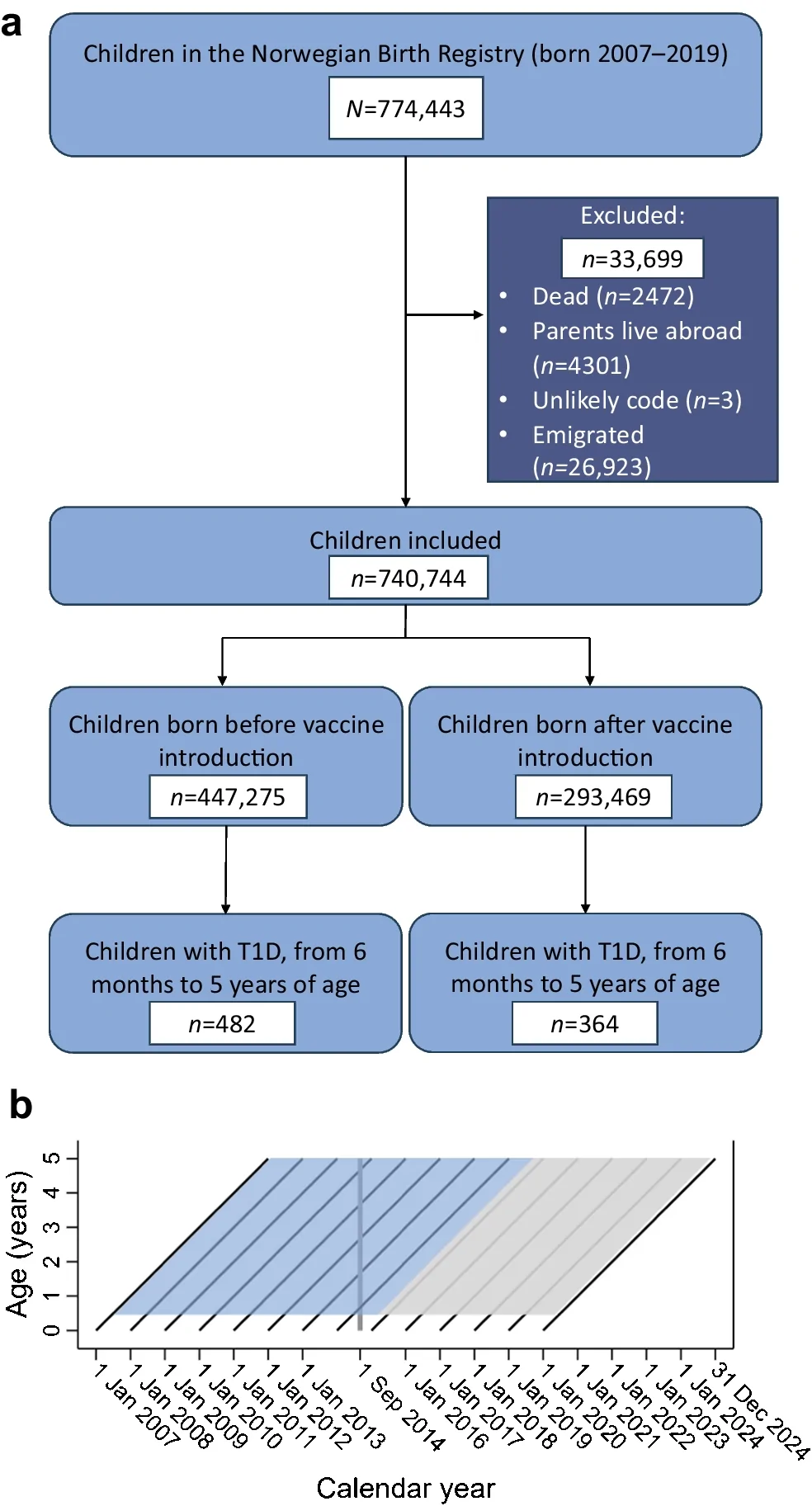
(**a**) Flow chart of included children born in Norway 2007–2019 in a population-based cohort. (**b**) Lexis diagram with calendar year on *x*-axis and age on *y*-axis. Diagonal lines indicate age for different birth years (2007–2019). Vertical line at 1 September 2014 marks rotavirus vaccine introduction. Blue field illustrates children born before rotavirus vaccine introduction and grey field children born after vaccine introduction. Follow-up was from 6 months of age to 5 years of age, to ensure similar distribution of follow-up for cohorts born before and after the vaccine introduction. T1D, type 1 diabetes

Rotavirus vaccination was introduced in the Norwegian Childhood Immunisation Programme in children born 1 September 2014 or later. This formed a natural experiment where we could compare type 1 diabetes incidence in comparable age-windows in the pre-vaccination and the post-vaccination cohort (Fig. 1). We conducted a nationwide children cohort study in Norway including children born 1 January 2007 to 31 December 2019, followed up until 31 December 2024. The study was approved by the Regional Committees for Medical Research Ethics, (REK Sør-Øst 2013/2114). As register data was used, personal consent was not required.

### Data sources

The following data sources were used:

- The Medical Birth Registry of Norway (MBRN) [13], which contains information about all births in Norway, and it is mandatory to register all pregnancies lasting longer than 12 weeks
- The Norwegian Patient Registry (NPR) [14], a mandatory registry since 2008, for all inpatient and outpatient consultations at public and private hospitals, clinics and contracts specialists; doctors register the diagnosis of the patient by the ICD-10 system, including E10 for type 1 diabetes
- The Norwegian Prescription Database (NorPD) [15], which contains information about dispensed drugs from pharmacies in Norway, including dispensed insulin (ATC code A10A)
- Statistics Norway (SSB) [16], which collects official individual-level information about the Norwegian population, such as information about maternal education, household income, country background and healthcare region
- The Norwegian Immunisation Registry [17], which is a register of all vaccines administered in Norway to people with a Norwegian birth number or a temporary unique identification number for people with a temporary stay in Norway; the number of rotavirus vaccine doses are included in this registry

### Study participants

To ensure comparability of groups before and after introduction of vaccination, we included children born 1 January 2007 to 31 December 2019 from the MBRN. Apart from month and year of birth we also received information on natal sex, vital status and emigration from the MBRN. We excluded children who were dead or had unknown living status, who had emigrated or whose parents were not permanently living in Norway (Fig. 1).

### Outcome

Individuals were defined as diagnosed with type 1 diabetes if at least one ICD-10 code for type 1 diabetes (E10) was entered in the NPR and insulin was dispensed from a pharmacy at least once (ATC code A10A) according to the NorPD. Children diagnosed late in 2024 might have been dispensed the first insulin from a pharmacy in 2024 but have a hospital discharge recorded in 2025, and therefore we defined all children with dispensed insulin in 2024 as having type 1 diabetes. Age at diagnosis was defined as the age at the first month in which insulin was dispensed. To ensure rotavirus vaccination before type 1 diabetes diagnosis and exclude potential monogenic diabetes, we started follow-up at 6 months of age. Three children were excluded due to type 1 diabetes before 6 months of age. For comparable age distributions in cohorts before and after introduction of the rotavirus vaccination programme, we ended follow-up at 5 years of age.

### Exposure

All children born in Norway after 1 September 2014 were, according to the National Childhood Immunisation Programme, offered rotavirus vaccination free of charge. The rotavirus vaccine used in Norway is RV1, which is a live-attenuated vaccine administered in two doses at 6 weeks and 3 months of age. The doses can be delayed but the first dose must not be administered later than 12 weeks of age and the second dose no later than 16 weeks of age according to the Norwegian guidelines [10]. Contraindications for rotavirus vaccine administration include ongoing infections with fever over 38°C, gastrointestinal malformation or previous intussusception, allergy to the vaccine, severe combined immunodeficiency and birth before gestational week 27 [10].

All children born after introduction of the rotavirus vaccination programme were considered exposed in the main analysis regardless of vaccination status. The pre- and post-vaccination cohorts were considered roughly equal regarding background risk factors in the main analysis (Table 1). In the secondary analysis on vaccination status, we compared fully vaccinated and partially vaccinated children with non-vaccinated children separately. In this analysis we excluded children with missing information about rotavirus vaccination (*n*=8) and those registered with more than four doses (*n*=10) due to likely errors in registration. For more detailed vaccination status by year of birth, see Table 2. For characteristics of non-vaccinated children, see Table 1.

**Table 1:** Characteristics of children included in the analysis born 2007–2019 in a Norwegian population-based cohort^a^

Characteristic	All	Born before vaccination implementation	Born after vaccination implementation	Non-vaccinated, born after vaccination implementation
No. of children, *n* (%)	740,744 (100)	447,275 (60.4)	293,469 (39.6)	12,586 (1.7)
Type 1 diabetes, *n* (%)^b^				
Yes	846 (0.1)	482 (0.1)	364 (0.1)	11 (0.1)
No	739,895 (99.9)	446,792 (99.9)	293,103 (99.9)	12,575 (99.9)
Rotavirus vaccination status, *n* (%)^c^				
Fully vaccinated	273,787 (37.0)	1418 (0.3)	272,369 (92.8)	0
Partially vaccinated	8807 (1.2)	301 (0.1)	8506 (2.9)	0
Non-vaccinated	458,132 (61.8)	445,546 (99.6)	12,586 (4.3)	12,586 (100)
Natal sex, *n* (%)^d^				
Male	380,636 (51.4)	229,540 (51.3)	151,096 (51.5)	6493 (51.6)
Female	360,108 (48.6)	217,735 (48.7)	142,373 (48.5)	6093 (48.4)
Household income, *n* (%)^e^				
Lowest quartile	184,773 (25)	106,671 (23.9)	78,102 (26.7)	4794 (38.3)
Second quartile	184,778 (25)	110,164 (24.7)	74,614 (25.5)	3148 (25.1)
Third quartile	184,769 (25)	111,771 (25.0)	72,998 (24.9)	2504 (20.0)
Highest quartile	184,771 (25)	117,646 (26.4)	67,125 (22.9)	2074 (16.6)
Maternal education, *n* (%)^f^				
Primary or lower	112,072 (15.4)	66,622 (15.0)	45,450 (16.0)	2143 (17.7)
Secondary education	189,815 (26.1)	122,418 (27.6)	67,397 (23.8)	3248 (26.8)
Higher education	424,195 (58.4)	253,776 (57.3)	170,419 (60.2)	6723 (55.5)
Healthcare region, *n* (%)^g^				
South-East	409,261 (55.3)	246,604 (55.2)	162,657 (55.5)	7093 (56.5)
West	167,461 (22.6)	100,967 (22.6)	66,494 (22.7)	2927 (23.3)
Mid	99,735 (13.5)	60,363 (13.5)	39,372 (13.4)	1211 (9.6)
North	63,724 (8.6)	39,161 (8.8)	24,563 (8.4)	1320 (10.5)
Country background, *n* (%)^h^				
Norwegian	461,804 (62.3)	293,491 (65.6)	168,313 (57.4)	6308 (50.1)
Other European	136,223 (18.4)	75,732 (16.9)	60,491 (20.6)	3966 (31.5)
Asian	78,880 (10.7)	43,406 (9.7)	35,474 (12.1)	1107 (8.8)
African	36,483 (4.9)	19,004 (4.3)	17,479 (6.0)	657 (5.2)
American	25,580 (3.5)	14,622 (3.3)	10,958 (3.7)	510 (4.1)
Oceania	1774 (0.2)	1020 (0.2)	754 (0.3)	38 (0.3)
Coeliac disease, *n* (%)^i^				
Yes	2795 (0.4)	1712 (0.4)	1083 (0.4)	32 (0.3)
No	737,949 (99.6)	445,563 (99.6)	292,386 (99.6)	12,554 (99.7)
Follow-up time, months, mean ± SD^j^	59.97±0.90	59.98±0.88	59.97±0.91	59.98±0.70

^a^A similar table has been published previously [11]

^b^Type 1 diabetes with follow-up starting at 6 months and ending at 5 years. Three children with type 1 diabetes before 6 months of age were excluded from all analyses. No missing data

^c^In addition, ten children with more than four registered rotavirus vaccine doses and eight with missing vaccination status

^d^No data missing

^e^Household income quartiles: lowest quartile <312,567 NOK; second quartile ≥312,567–<395,776.67 NOK; third quartile ≥395,776.67–<488,374 NOK; highest quartile ≥488,374 NOK. In total 1653 had missing household income, of which 1023 were born before vaccination implementation and 630 after. Of children born after vaccination implementation and not vaccinated, 66 had missing household income

^f^A total of 14,662 had missing data on maternal education, of which 4459 were born before vaccination implementation and 10,203 after. Of children born after vaccination implementation and not vaccinated, 472 had missing maternal education

^g^Norway has four health regions based on geographic location. A total of 563 had missing data on healthcare region, of which 180 were born before vaccination implementation and 383 after. Of children born after vaccination implementation and not vaccinated, 35 had missing healthcare region

^h^For children born in Norway, this is the parents’ country of birth. If the country of birth differed between parents, the maternal country background was chosen. If both parents were born in Norway the third-generation’s country of birth was chosen in the following order: mother of the mother; father of the mother; mother of the father; and father of the father. No missing data

^i^Coeliac disease at 5 years of follow-up. No missing data

^j^Mean follow-up time for main analysis. Maximal follow-up time for main analysis was 60 months (5 years)

**Table 2:** Vaccination status by year of birth before and after vaccination implementation in September 2014

Year of birth	Non-vaccinated, *n* (%)	Fully vaccinated, *n* (%)	Partially vaccinated, *n* (%)	Total, *n*^a^
2007–2013	407,314 (99.8)	680 (0.2)	166 (0.04)	408,160
2014 Jan–Aug	38,232 (97.8)	738 (1.9)	135 (0.3)	39,105
2014 Sep–Dec	1723 (9.9)	15,119 (86.7)	603 (3.5)	17,445
2015	3193 (5.6)	51,727 (91.4)	1666 (2.9)	56,586
2016	2761 (4.8)	52,410 (92.0)	1825 (3.2)	56,996
2017	1818 (3.3)	51,565 (93.7)	1628 (3.0)	55,011
2018	1590 (3.0)	50,863 (94.5)	1359 (2.5)	53,812
2019	1501 (2.8)	50,685 (94.5)	1425 (2.7)	53,611

^a^Data for a total of 740,726 children are presented in this table, 18 children were excluded due to missing vaccination status or having more than four registered doses of rotavirus vaccine

### Other variables

In the secondary analysis on vaccination status, we adjusted for country background, birth year, household income, maternal education, natal sex and healthcare region of residence. All children in the study were born in Norway, and country background was defined as the country of birth of the parents and grandparents [18]. Maternal country of birth was chosen if it was different from that of the father. If both parents were born in Norway, country background was defined as the country of birth for the grandparents, in the following order among grandparents if not the same for all: mother of the mother; father of the mother; mother of the father; and father of the father. We regarded all the abovementioned variables as possible confounders in the secondary Cox regression analyses on vaccination status. Children with missing adjustment variables were excluded from adjusted analysis. Most covariates were provided from SSB [16], except for birth year and natal sex, which were provided from the MBRN. We also received data on ICD-10 codes for other specified diabetes mellitus (E13) and unspecified diabetes mellitus (E14) from the NPR (these codes are sometimes used together with E10 [type 1 diabetes]) and used this information to exclude diabetes cases that were potentially monogenic in a sensitivity analysis (see below).

### Data analysis

For all analyses we used Stata 17 (StataCorp LLC, College Station, TX, USA). For the main analysis we used interrupted time series comparing incidence rates of type 1 diabetes by birth before compared with after introduction of the rotavirus vaccine programme, using Cox regression to investigate whether there was a significant break in time trend [19]. This analysis accounts for underlying long-term loglinear trends in incidence by time of birth.

In a secondary analysis we estimated HRs with 95% CIs, comparing the hazard of type 1 diabetes among those fully vaccinated (with two or more doses of vaccine) and partially vaccinated (one dose) with non-vaccinated children, regardless of birth year, with and without adjustment for possible confounders mentioned earlier.

We did a series of other a priori and post hoc sensitivity analyses to assess the robustness of our main result towards various assumptions and definitions. These were done with the primary analysis model using interrupted time series and changing one factor at a time.

First, while we ended follow-up at the age of 5 years in our primary analyses to ensure equal follow-up for everybody before and after introduction of vaccination, we also did analyses ending follow-up at the age of 4, 6 or 7 years (excluding children born in 2018 and 2019 in the latter to have comparable age groups) (Fig. 1). We hypothesised that there could be herd immunity among children close in age around the year in which the vaccination programme was introduced. Therefore, in a sensitivity analysis, we excluded children born 1 year before to 1 year after rotavirus vaccination introduction (2013–2015).

Second, we excluded children with potential monogenic diabetes, defined as children who were regarded as having type 1 diabetes in the main analysis but in addition were registered with ICD-10 code E13 (other specific types of diabetes) or E14 (unspecific type of diabetes) in both 2023 and 2024.

**Fig. 2:**
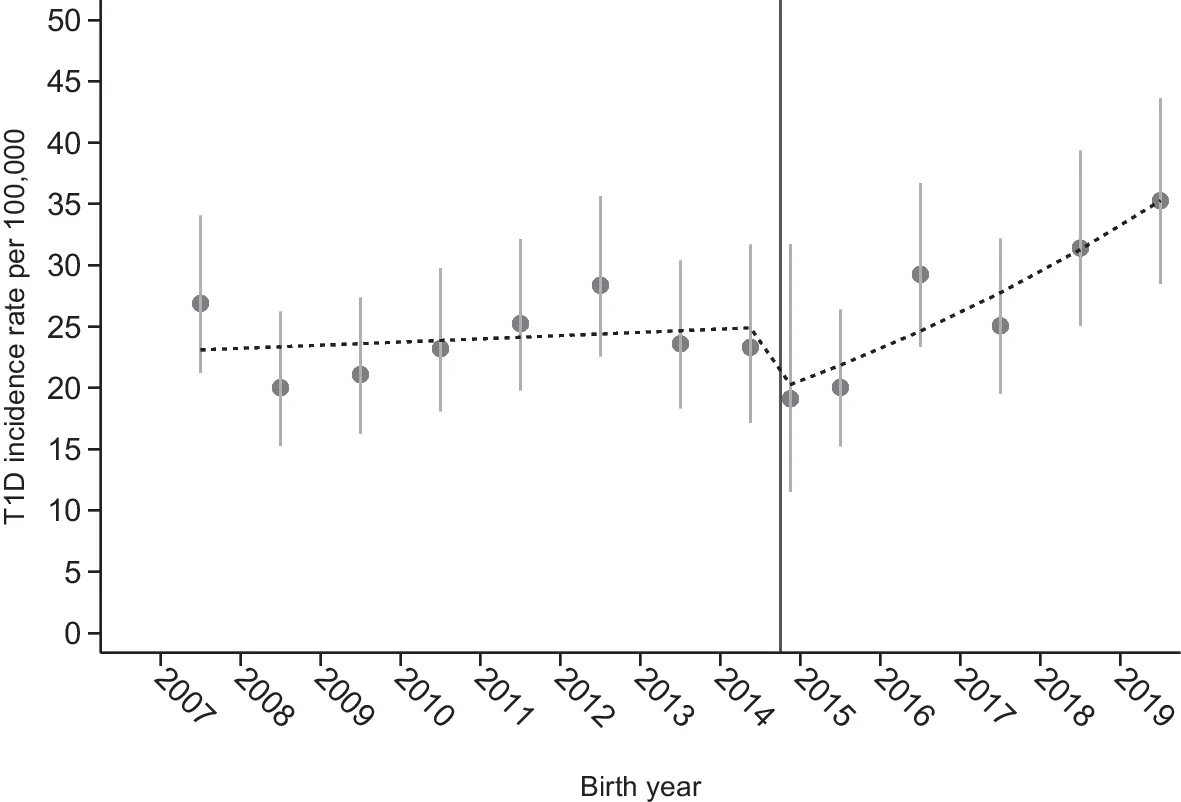
Incidence rate (95% CI) of type 1 diabetes in children aged 6 months to 5 years by birth year before and after introduction of rotavirus vaccine in the National Childhood Immunisation Programme in Norway. The vertical line indicates the introduction of rotavirus vaccine on 1 September 2014. The relative change in trends from the interrupted time series Cox regression was 1.11 (95% CI 1.03, 1.20; *p*=0.008). T1D, type 1 diabetes

Third, we did two post hoc interrupted time series analyses to assess the potential effect of other changes in the childhood vaccination programme in Norway during the study period. In the first of these, we analysed the introduction of the hepatitis B vaccine to all children born on 1 November 2016 or later, to investigate whether our results could be due to underlying temporal changes and not restricted to the rotavirus vaccine. In the second of these analyses, as a form of negative control, where we assumed no difference in type 1 diabetes incidence of change from the 7-valent to the 13-valent conjugated pneumococcal vaccine on 1 April 2011, we similarly compared type 1 diabetes incidence in children born before and children born after this change. Both the hepatitis B and the pneumococcal vaccines are administered in three doses at 3, 5 and 12 months of age. In the pneumococcal vaccine analysis, we used births from 1 November 2010 as the cut off, as children born at this date or later were offered at least two of the three pneumococcal vaccine doses in the form of the 13-valent version (see electronic supplementary material [ESM] Figs 1, 2).

## Results

A total of 740,744 children were included in the cohort (Fig. 1). Of these, 3256 developed type 1 diabetes at a median age of 7.8 years by the end of 2024. A total of 846 children developed type 1 diabetes at the age of 6 months to 5 years. In children born 1 September 2014 to 31 December 2019 (after introduction of the rotavirus vaccination programme), 92.8% were fully vaccinated, compared with 0.3% of those born before general vaccination introduction (Table 1). Only 1418 children were fully vaccinated and 301 partially vaccinated before general vaccine introduction (Table 2). In the whole cohort 37.0% were fully vaccinated, 1.2% partially vaccinated and 61.8% non-vaccinated (Table 1).

The main analysis, using interrupted time series, indicated an increasing trend of type 1 diabetes incidence in cohorts born after the introduction of rotavirus vaccination (HR 1.11 [95% CI 1.03, 1.20], *p*=0.008; Fig. 2).

In the secondary analysis on fully vaccinated compared with non-vaccinated children the adjusted HR was 1.17 (95% CI 0.78, 1.77) (Table 3).

**Table 3:** Risk of type 1 diabetes comparing fully vaccinated and partially vaccinated children with non-vaccinated children in a Norwegian population-based cohort with follow-up from 6 months to 5 years of age by Cox regression analysis

Vaccination status	Total	T1D	HR (95% CI)	Adjusted HR (95% CI)^a^
No. of children	740,726^b^	846		
Non-vaccinated	458,131	490	1.0 ref.	1.0 ref.^c^
Partially vaccinated	8807	11	1.17 (0.64–2.12)	1.29 (0.64–2.62)^d^
Fully vaccinated	273,785	345	1.18 (1.03–1.35)	1.17 (0.78–1.77)^e^

^a^Adjusted for maternal education, household income, country background, healthcare region, natal sex and year of birth; in the analysis 2.2% did not have complete data and were therefore excluded from adjusted analysis

^b^Eighteen children were excluded due to missing vaccination status or more than four registered doses of rotavirus vaccine

^c^Adjusted analysis included 724,325 children with 485 non-vaccinated individuals with type 1 diabetes

^d^Adjusted analysis included 11 partially vaccinated children; unadjusted HR in the same sample was 1.25 (95% CI 0.69, 2.26)

^e^Adjusted analysis included 337 fully vaccinated children; unadjusted HR in the same sample was 1.19 (95% CI 1.04, 1.37)

T1D, type 1 diabetes

### Sensitivity analyses

In the sensitivity analyses to assess the influence of having a shorter or longer follow-up time, results did not change appreciably compared with those of the primary 5 year follow-up, although the result was not statistically significant with a 7 year follow-up (Table 4).

**Table 4:** Sensitivity analyses using interrupted time series estimating relative HR with 95% CI in children born after compared with before rotavirus vaccination implementation

Analysis	Total, *n*^a^	Individuals with T1D born before vaccine implementation, *n* cases	Individuals with T1D born after vaccine implementation, *n* cases	Relative HR (95% CI)	*p* value
Excluding children born 2013–2015 to assess potential herd immunity	571,053	381	298	1.05 (0.93, 1.18)	0.42
Ending follow-up at age 4 years to assess the influence of shorter follow-up	740,741	349	258	1.17 (1.07, 1.29)	0.001
Ending follow-up at age 6 years to assess the influence of longer follow-up	740,741	644	480	1.09 (1.01, 1.16)	0.02
Ending follow-up at age 7 years to assess the influence of even longer follow-up^b^	633,318	815	383	1.03 (0.92, 1.15)	0.60
Excluding children with potential monogenic diabetes^c^	740,737	480	362	1.11 (1.03, 1.20)	0.009

^a^Due to follow-up starting at 6 months of age, three children with type 1 diabetes before 6 months of age were excluded from the total number of children

^b^Children born in 2018 and 2019 were excluded from analysis of the 7 years follow-up to ensure equal distribution of follow-up in the group born before and after rotavirus vaccination introduction

^c^Four children defined as having type 1 diabetes in the main analysis but also registered with ICD-10 codes E13 in both 2023 and 2024 (*n*=2) or E14 in both 2023 and 2024 (*n*=2) were excluded

T1D, type 1 diabetes

When excluding children born in 2013–2015, possibly affected by herd immunity, the relative HR for type 1 diabetes was 1.05 (95% CI 0.93, 1.18; *p*=0.42; Table 4).

When excluding four children with potential monogenic diabetes the result did not differ appreciably from that of the main analysis (Table 4).

Our post hoc analyses, using the same type of statistical analysis to study other changes in the vaccination programme, showed non-significant upward trends in type 1 diabetes incidence after the introduction of the hepatitis B vaccine on 1 November 2016, (relative HR 1.10 [95% CI 0.96, 1.28]; *p*=0.18; see ESM Fig. 1 and ESM Table 1), and after changing from 7-valent to 13-valent pneumococcal vaccine in 2011 (relative HR 1.10 ([95% CI 0.97, 1.24]; *p*=0.13; see ESM Fig. 2 and ESM Table 1).

## Discussion

Our findings in this natural experiment of rotavirus vaccine implementation in the National Childhood Immunisation Programme in September 2014 indicate a break in trend, with increased type 1 diabetes incidence rate after rotavirus introduction, opposite to the hypothesised direction. However, a range of sensitivity analyses suggests that the primary observation may not reflect causality.

### Strengths and limitations

Our study had a large study sample with few exclusions. We had the opportunity to compare type 1 diabetes in the whole cohort born before vaccination implementation with the whole cohort born after implementation in an interrupted time series analysis, and also to compare vaccinated with non-vaccinated children. A strength of the interrupted time series analysis is that underlying trends that occur gradually, such as could be the case for risk factors, are accounted for [19]. The method measures sudden break in trend, as could be expected if suddenly removing or adding a risk factor [19]. The basic premise of the interrupted time series analysis is to compare the projection of the time trend before the introduction of the rotavirus vaccine programme with the observed time trend after the rotavirus vaccination implementation [19]. The high vaccination rate after implementation of the rotavirus vaccination programme and the very limited use before implementation brings contrasts, close to a natural experiment. Access to dispensed insulin data gave us valuable validation of type 1 diabetes diagnosis. As children with monogenic diabetes also sometimes use insulin, we excluded children with potential monogenic diabetes in a sensitivity analysis. We were able to have similar distribution of age in the pre- and post-rotavirus vaccination introduction cohorts. Since the incidence of type 1 diabetes increases strongly with age during the first few years of life, a shorter follow-up time of later birth cohorts compared with earlier birth cohorts might artificially bias the observed associations towards an inverse association between rotavirus vaccination and risk of type 1 diabetes. In total, 101 children had both coeliac disease and type 1 diabetes by the age of 5 years. Only 22 individuals had coeliac disease before type 1 diabetes and therefore adjustment by coeliac disease was not a feasible additional analysis.

### Comparison with existing literature

A recent review and analysis of ecological data from eight countries concluded that there was a decrease in type 1 diabetes incidence among children aged under 5 years in seven of the countries after introduction of rotavirus vaccination in the respective Childhood Immunisation Programmes, a result contrary to ours [20]. The ecological design in this published review did not allow a clear separation by birth cohort, nor did it provide data on vaccination status at an individual level as we had in our data [20]. Therefore, the articles included in the review [20] could neither compare type 1 diabetes in vaccinated and non-vaccinated children nor separate data for RV1 and the pentavalent rotavirus vaccine RV5 when both vaccines were used. There were two studies using RV1 that showed a reduction in type 1 diabetes incidence, one from Ireland [21] and one from Wales [22]. These two studies, and other studies from countries where both RV1 and RV5 were used, demonstrated a more modest reduction in the incidence of type 1 diabetes than when RV5 was used alone [20–22].

A recent meta-analysis by Kosmeri et al [23] excluded ecological studies and six studies with individual-level data that the authors judged to have high risk of bias. Of the five included studies, three were based on commercial databases in the USA. The pooled HR was 0.87 (95% CI 0.78, 0.98), largely driven by a single study by Rogers et al [24]. Meta-analyses are prone to publication bias and judgements regarding inclusion of studies. This meta-analysis included only five studies with signs of moderate heterogeneity. Not all included studies in the meta-analysis separated fully vaccinated from partially vaccinated individuals [25, 26] and several studies did not separate the types of rotavirus vaccines in use [24, 27, 28]. Furthermore, some studies only compared non-vaccinated children with vaccinated children after rotavirus vaccine implementation [27, 28], which can be problematic due to herd immunity against rotavirus infection.

Only one study, by Rogers et al [24], had a statistically significant finding of reduced likelihood for type 1 diabetes in fully vaccinated children. Of vaccinated children, 83.3% were vaccinated with RV5 and the rest with RV1. Although RV5 reduced the likelihood of type 1 diabetes by 37%, RV1 did not significantly reduce type 1 diabetes, with an HR of 0.90 (95% CI 0.62, 1.31) when modelled separately. This provides an opening for a discussion of whether different rotavirus vaccines have different associations with type 1 diabetes. RV5 is a three-dose pentavalent reassortant human-bovine vaccine expressing the VP7 protein of subtypes G1, G2, G3, G4, G6 as well as the VP4 P7[5] and P1A[8] [29]. The RV1 vaccine is a two-dose monovalent vaccine derived from the P1[8]G1 human strain [29].

Of the other studies included by Kosmeri et al, two American studies [27, 28] failed to find any significant association between rotavirus vaccine and type 1 diabetes. Burke et al [28] did not distinguish between RV1 and RV5, while Glanz et al [27], with a subanalysis of RV1 data, found an adjusted HR of 2.03 (95% CI 0.66, 6.28).

The study by Inns et al [25] in the UK is quite similar to our study in terms of sample size and methods. RV1 is also used in the UK, but Inns et al did not analyse partially and fully vaccinated children separately. The HR for type 1 diabetes was 0.89 (95% CI 0.68, 1.19) with a 7 year follow-up.

The last included study in the meta-analysis by Kosmeri et al was from Finland, by Vaarala et al [26]. They used RV5 exclusively but had a limited sample size and only included children born in 2009–2010. Of note, 37.7% were already vaccinated before implementation of the National Childhood Immunisation Programme. Considering that the programme started in children born on 1 July 2009, this study could be biased by herd immunity, and an interrupted time series analysis could not be performed.

The systematic review by Burke et al [7] is largely similar to that of Kosmeri et al but did not include the study by Inns et al [25] and included instead a Finnish study by Hemming-Harlo et al [30]. Hemming-Harlo et al found no difference in type 1 diabetes prevalence in RV5-vaccinated children compared with placebo recipients, with an 11–14 year follow-up. There were few cases in total and only 30% follow-up of the whole randomised controlled vaccine trial.

### Potential explanations and implications

While the incidence of type 1 diabetes has been increasing over several decades, many high-incidence countries have shown signs of levelling off since the mid-2000s with no immediate explanation [3]. Introduction of rotavirus vaccines is a plausible explanation for the flattening incidence curves in some countries and although this has been supported by some studies discussed above, the studies have not all been consistent. We cannot exclude that unknown factors linked to type 1 diabetes risk may have changed concomitantly with introduction of the rotavirus vaccine in Norway. We are, however, not aware of any such plausible factors but discuss some speculative alternatives below.

After the COVID-19 pandemic, many countries experienced an increase in the incidence of type 1 diabetes [31]. In Norway, there was a temporary increase in type 1 diabetes incidence in 2022 followed by a decrease [32]. However, a pooled analysis of Swedish and Norwegian adolescents did not find an increase in type 1 diabetes incidence after COVID-19 infection [33]. We cannot rule out changes due to the pandemic that could have influenced type 1 diabetes incidence but there is no clear association between SARS-CoV-2 and type 1 diabetes, and very few children aged <5 years received a SARS-CoV-2 vaccine [33].

In June 2018, the Norwegian prenatal follow-up guidelines started recommending measurement of ferritin concentration in serum in the first trimester of pregnancy to determine the need for iron supplement [34]. In 2006–2018 the recommendation was measurement of haemoglobin in week 30 of the pregnancy, which is a less sensitive method [35]. High iron levels are suspected to be associated with type 1 diabetes [35]. We, however, do not think this is an issue for our study since only children born in 2019 would be affected and iron levels are usually low in pregnancy.

There have not been any changes in type 1 diabetes diagnostics during the study period that we are aware of and there is so far no nationwide type 1 diabetes screening in Norway. There have been changes in the Norwegian Childhood Immunisation Programme during the study period. Hepatitis B vaccination was introduced to children born 1 November 2016 or later, administered in three doses up to 1 year of age [36]. The pneumococcal vaccine was changed from the 7-valent (used 2006 to April 2011) to the 13-valent vaccine [37], administered at the same time as the hepatitis B vaccine. Previously the tuberculosis vaccine was given to all children in their teens but since 2009 the vaccine is given at 6 weeks of age but only to children at high risk of tuberculosis, whose parents come from a country with high prevalence of tuberculosis [36]. The Pandemrix vaccine was used in children and adults starting 1 October 2009 to 10 August 2010 [38] but was not included in the national vaccination programme. However, there is no previously known association between these vaccines and type 1 diabetes.

The additional subanalyses did not find a significant change in trend for the change of pneumococcal vaccine or introduction of the hepatitis B vaccine. However, although not significant, the fact that the point estimate for the relative increase in incidence rate of type 1 diabetes after both introduction of the hepatitis B vaccine and change of the pneumococcal vaccine were of the same magnitude as that for rotavirus vaccine indicates that our main rotavirus vaccine results should be interpreted with caution.

In our dataset, partially vaccinated children were few and the results should therefore be interpreted cautiously. We did not have information on why some children only received one dose of the rotavirus vaccine, although they might be a selected group that for example received the first dose of vaccine but then were found to have contraindications for the second dose.

Rotavirus vaccines are live vaccines administered orally and induces an immune-response by replicating in the gut without causing severe symptoms [12]. If rotavirus infection is associated with type 1 diabetes, the vaccine could in theory act the same way. In our study we used RV1 and the results are therefore not generalisable to countries using other rotavirus vaccines. It is important to highlight the huge impact that rotavirus vaccination has had on reducing child mortality globally as well as hospitalisation due to rotavirus infection [5, 39].

### Conclusion

In conclusion, while our primary analysis showed an increased type 1 diabetes incidence rate below age 5 years after rotavirus vaccination introduction in Norway, a range of sensitivity analyses suggests that the primary observation may not reflect causality. We conclude that our data do not support the hypothesis that rotavirus vaccination reduces the incidence of type 1 diabetes. Future research on this topic is needed in different settings, separating different types of rotavirus vaccines.

## Supplementary Information

Below is the link to the electronic supplementary material.

ESM (PDF 183 KB)

## Data Availability

Because of data protection legislation, the authors are not permitted to make data available to others. However, the same data can be made available to researchers by applying independently to the data owners. This requires submitting applications via https://www.ssb.no/en/data-til-forskning/utlan-av-data-til-forskere/soknad-offentlig-myndighet (data from SSB) and https://helsedata.no/ (all other data; registries managed by the Norwegian Institute of Public Health). Acceptance requires approval from the Regional Committees for Medical and Health Research Ethics in Norway and compliance with the EU General Data Protection Regulation.

## Abbreviations

MBRN: Medical Birth Registry of Norway
NorPD: Norwegian Prescription Database
NPR: Norwegian Patient Registry
RV1: Rotarix monovalent oral rotavirus vaccine
RV5: RotaTeq pentavalent oral rotavirus vaccine
SSB: Statistics Norway
VP: Virus protein
