# Supplementary material for: Rotavirus vaccination and incidence of type 1 diabetes: a population-based natural experiment
Source: Diabetologia. 2026 Aug 28;69(12):3559–69. doi: 10.1007/s00125-026-06832-0 (PMC13714045; doi:10.1007/s00125-026-06832-0)
Supplement: Supplementary file 1 [file 125_2026_6832_MOESM1_ESM.pdf]

ESM: table 1 Sensitivity analyses with interrupted time series analysis estimating hazard ratio with 95% confidence interval in children born after compared to children born before changes in the Norwegian childhood immunization programme with follow-up from 6 months to 5-years age.

Analysis	Total, *n*[a]	Born before vaccine introduction, *n* (cases)	Born after vaccine introduction, *n* (cases)	HR (95% CI) *p* value
Risk of type 1 diabetes if born after compared to before hepatitis B vaccine introduction (1st of November 2016)	740,741	609	237	1.10 (0.96-1.28) *p*=0.18
Risk of type 1 diabetes if eligible for two or more doses of the new 13-valent pneumococcal vaccine (children born after 1st of November 2010)	740,741	229	617	1.10 (0.97-1.24) *p*=0.13

[a] Three children with type 1 diabetes before 6 months of age are excluded from total number of children.

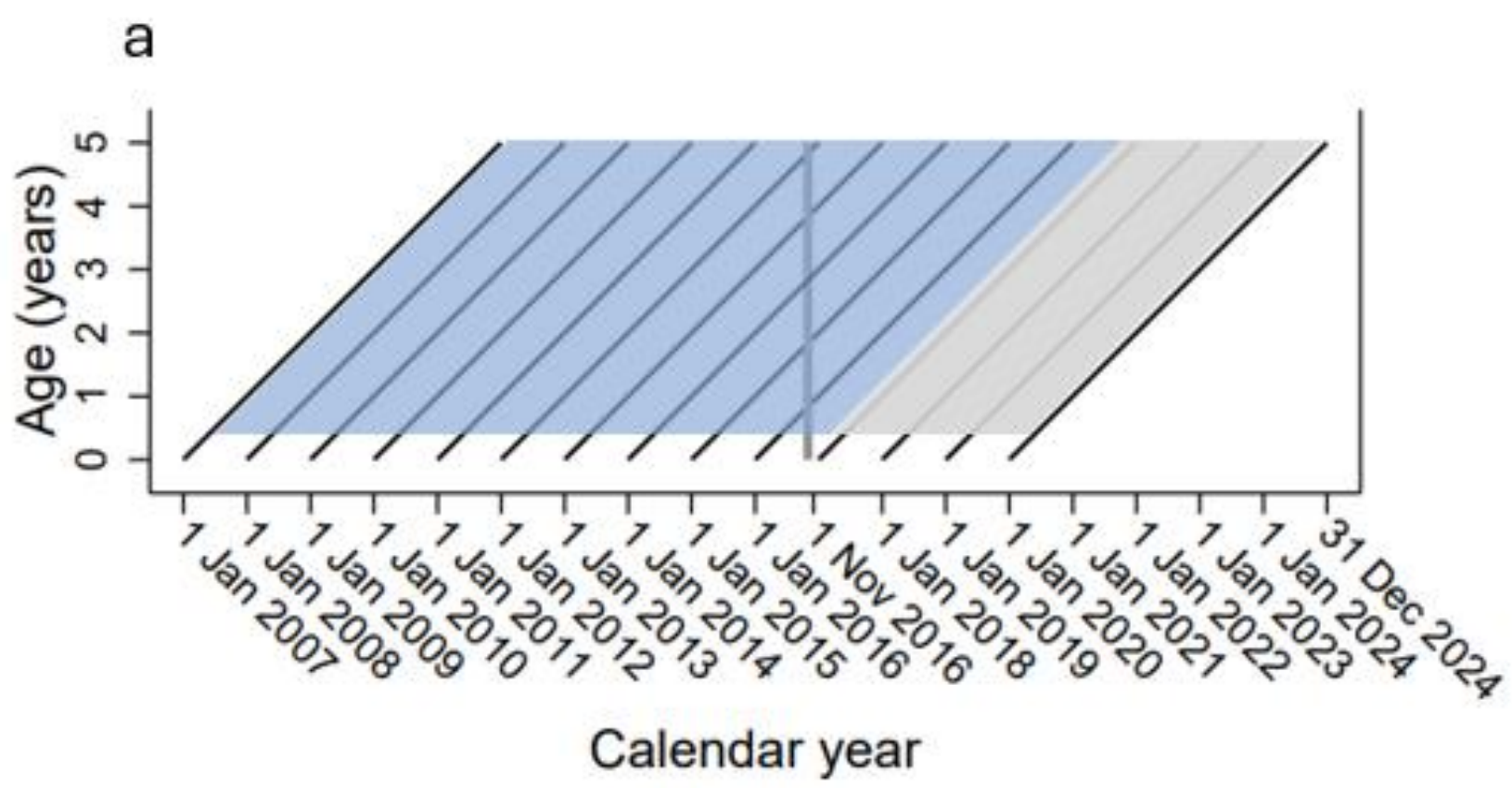

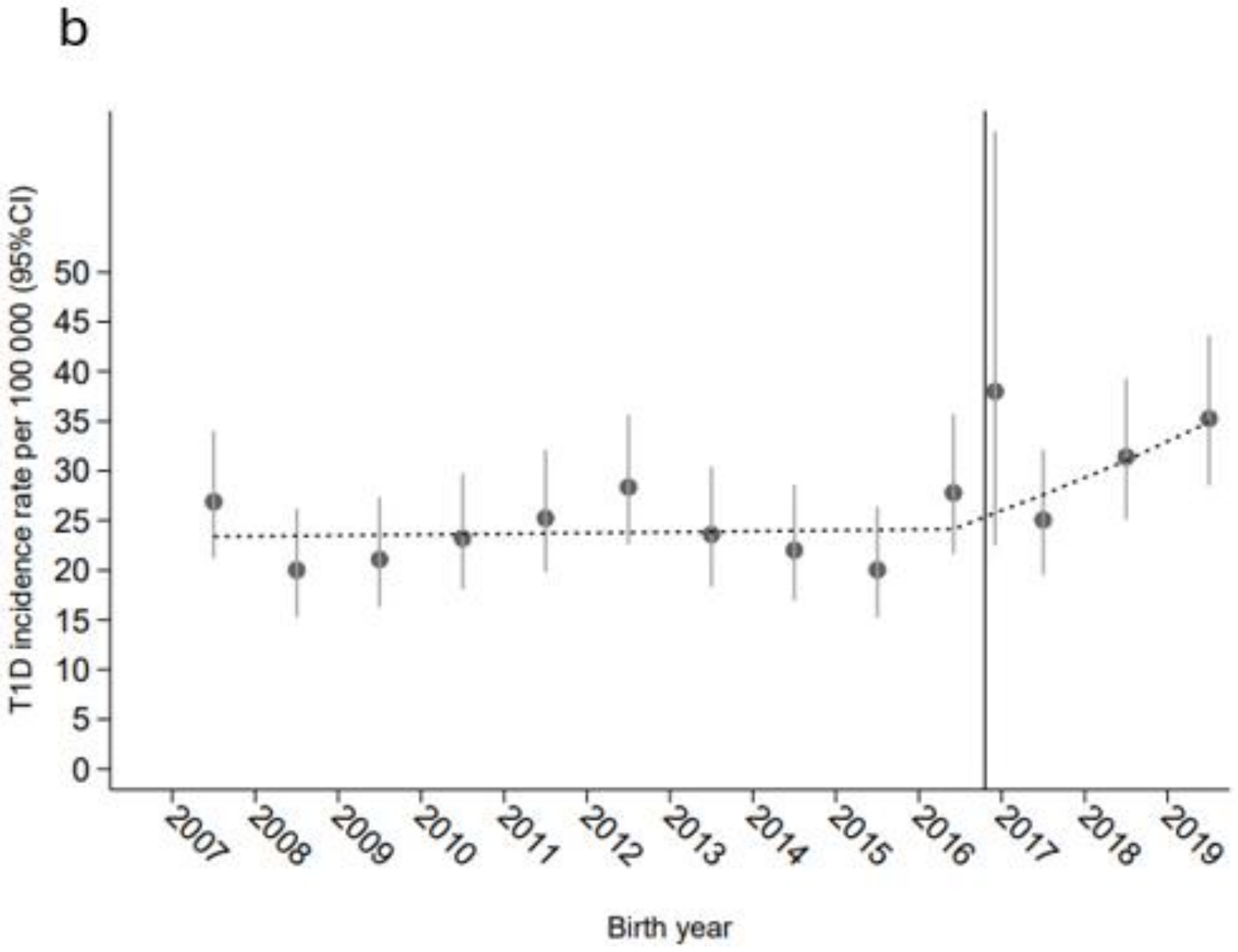

ESM: Fig. 1

- **a.** **Lexis diagram with calendar year on x-axis and age on y-axis.** Diagonal lines indicate age for different birth years (2007-2019). Vertical line at 1 November 2016 marks hepatitis B vaccine introduction. Blue field illustrates children born before hepatitis B vaccine introduction and grey field children born after hepatitis B vaccine introduction. Follow-up was from 6 months of age to 5 years of age.
- **b.** **Incidence rate (95% CI) of type 1 diabetes in children aged 6 months to 5 years by birth year before and after introduction of hepatitis B vaccine in the Norwegian Childhood Immunisation Programme.** The vertical line indicates the introduction of hepatitis B vaccine on 1 November 2016. The relative change in trends from the interrupted time series Cox regression was 1.10 (95% CI 0.96,1.28; *p*=0.18). T1D, type 1 diabetes

a

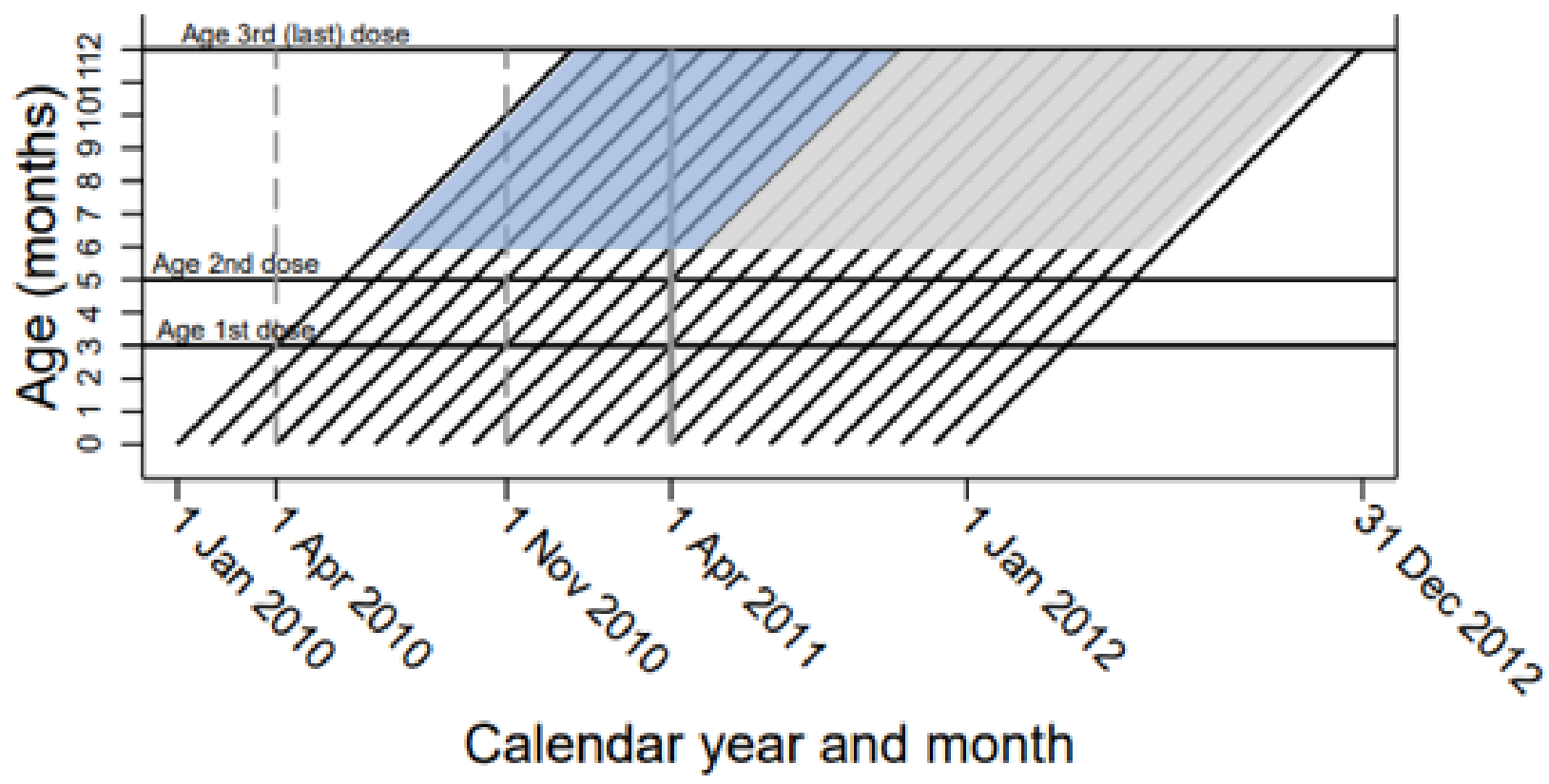

b

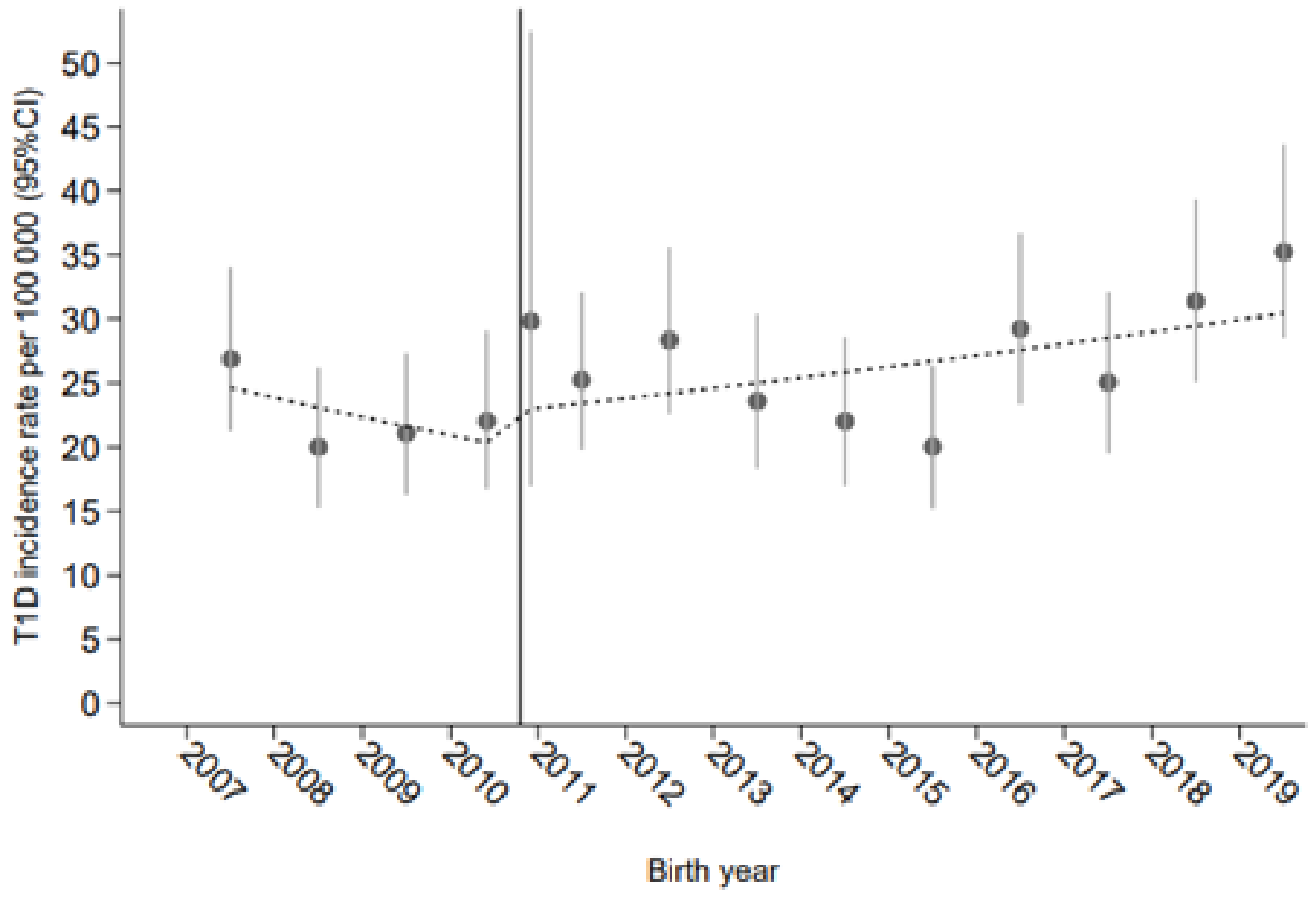

ESM: Fig. 2

a. **Lexis diagram with birth months on x-axis and age on y-axis.** Diagonal lines indicate age for different birth months (1 January 2010 to 31 December 2012). Vertical line at 1 April 2011 marks change of pneumococcal vaccine from 7-valent to 13-valent for all children. Pneumococcal vaccine is administered at 3-, 5- and 12 months of age. Blue field illustrates children who might have received one dose of the new 13-valent pneumococcal vaccine (born before 1 November 2010), considered not exposed in our analysis. The grey field illustrates children who might be exposed for two or three doses of the 13-valent vaccine (born 1 November 2010 or later), considered exposed in our analysis. Follow-up time from 6 months of age.
b. **Incidence rate (95% CI) of type 1 diabetes in children aged 6 months to 5 years by birth year before and after change of pneumococcal vaccine from 7-valent to 13-valent in the Norwegian Childhood Immunisation Programme.** As indicated by the vertical line on 1 November 2010, we considered children eligible for two doses of the 13-valent vaccine exposed. The relative change in trends from the interrupted time series Cox regression was 1.10 (0.97,1.24; p=0.13). T1D, type 1 diabetes
